# Low expression and Hypermethylation of ATP2B1 in Intrahepatic Cholangiocarcinoma Correlated With Cold Tumor Microenvironment

**DOI:** 10.3389/fonc.2022.927298

**Published:** 2022-07-07

**Authors:** Xiehua Zhang, Yuchao He, Peiqi Ren, Lu Chen, Zhiqiang Han, Lisha Qi, Liwei Chen, Yi Luo, Ning Zhang, Wei Lu, Hua Guo

**Affiliations:** ^1^ Department of Tumor Cell Biology, Tianjin Medical University Cancer Institute and Hospital, National Clinical Research Center for Cancer, Key Laboratory of Cancer Prevention and Therapy, Tianjin’s Clinical Research Center for Cancer, Tianjin, China; ^2^ Department of Hepatobiliary Oncology, Liver Cancer Research Center, Tianjin Medical University Cancer Institute and Hospital, National Clinical Research Center for Cancer, Key Laboratory of Cancer Prevention and Therapy, Tianjin's Clinical Research Center for Cancer, Tianjin, China; ^3^ Department of Infectious Diseases, The First Affiliated Hospital of Baotou Medical College, Baotou, China; ^4^ Department of Pathology, Tianjin Medical University Cancer Institute and Hospital, National Clinical Research Center for Cancer, Key Laboratory of Cancer Prevention and Therapy, Tianjin’s Clinical Research Center for Cancer, Tianjin, China

**Keywords:** ATP2B1, intrahepatic cholangiocarcinoma, immune subtypes, Ca^2+^, treatment

## Abstract

**Background:**

The efficacy of current therapeutic schedule is limited owing to fibroproliferative tumor microenvironment (TME) of cholangiocarcinoma, compelling a search for new therapeutic targets.

**Methods:**

Gene expression profiles and methylation profiles were obtained from UCSC Xena. Consensus clustering was performed on the transcriptome data of cholangiocarcinoma to determine the different immune subtypes. The differentially expressed genes (DEGs) between hot tumor and cold tumors were identified. ESTIMATE was used to assess immune score, and the cases were separated into relatively superior and inferior immune score groups. Single-sample gene set enrichment analysis was applied to assess 28 immune cells in the cholangiocarcinoma microenvironment. Unsupervised consensus was applied for methylation profiling to distribute the high and low methylation groups. The correlation between DNA methylation and mRNA expression was investigated, and the relationship between the *ATP2B1* gene and the immune microenvironment was explored. Finally, 77 cases of intrahepatic cholangiocarcinoma (ICC) were collected for verification.

**Results:**

Seven subtypes were related to patient outcomes (*P*=0.005). The proportions of CD8^+^ T cells in the “hot” immune type was significantly greater than that in the “cold” immune type (*P*<0.05). Next, DEGs and DNA methylation-governed genes were intersected, and *ATP2B1* was identified as a prognosis factor in ICC (*P*=0.035). ATP2B1 expression was positively correlated with immune scores (*P*=0.005, r=0.458), the levels of infiltrating CD8^+^ T cells (*P*=0.004, r=0.47), and CD4^+^ T cells (*P*=0.027, r=0.37). Immunohistochemistry confirmed that the amounts of CD8^+^ and CD4^+^ T cells were significantly higher in ICC tissue samples than in tissues with ATP2B1 overexpression (*P*<0.05).

**Conclusions:**

ATP2B1 overexpression can activate immune signals and prompt cold tumor response.

## Introduction

Cholangiocarcinoma is the second most common primary malignant liver tumor. The incidence rate and mortality rates of cholangiocarcinoma, particularly intrahepatic cholangiocarcinoma (ICC), have increased since 2000 (1). Although surgery is the only potential cure, most patients (∼70%) are diagnosed at late stages because of the lack of specific symptoms, and lose the opportunity of surgery. For some patients who underwent surgical resection, about 20% still had a very early recurrence within 6 months (2). Therefore, the development of effective systemic therapies for nearly every patient with ICC is increasing needed.

To date, cancer immunotherapy has achieved a satisfactory curative effect hepatocellular carcinoma (HCC) treatment (3). However, cholangiocarcinoma is characterized by a fibroproliferative TME, including cancer-associated fibroblasts, and immune cells (tumor-associated macrophages, neutrophils, and tumor-infiltrating lymphocytes). The fibroproliferative TME limits the efficacy of the current therapeutic schedule. The tumor immune microenvironment of different patients with the same cancer type is diverse. A study based on the TME of ICC confirmed that 45% of ICCs displayed an immune desert type (immune-suppressing tumor, cold tumor), and only 11% of the inflammatory type (immune-infiltrating tumor, hot tumor) showed a large number of lymphocyte infiltration, and was connected with the longest survival time of patients. The TME was simply classified as cold and hot tumors based on the level of proinflammatory cytokine production and T cell infiltration (4). Cold tumors are characterized by decreased cytotoxic lymphocyte infiltration at the tumor margin or in fibrous areas, and accompanied by a large number of Tregs, M2 macrophages and myeloid-derived suppressor cells (MDSCs) (5). Cold tumors cannot produce spontaneous immune infiltration and benefit from immunotherapy, due to lack of antigenicity or immune prototype (6). On the contrary, hot tumors not only contain highly infiltrated CD8^+^ T lymphocytes cells and M1 macrophages, but also low infiltrated MDSCs (5). In addition, some chemokines can recruit lymphocytes. For the infiltration of corresponding T cells, hot tumors will up regulate the immune checkpoints in TME (6). Although PD-L1 is elevated in about 60% of cholangiocarcinoma cases, the efficacy of immune checkpoint blockade is disappointing (7, 8). The low efficacy is related to the fact that Kupffer and dendritic cells in the TME secrete PD-L1 and are utilized by the tumor, which further leads to T cells depletion (9). The research confirmed that the TME hinders T cells proliferation and activation, and thus limits the benefits of immunotherapy. Therefore, exploring the whole TME and individualized treatment according to different environments is important. TME classification plays an important role in predicting the curative effect on cholangiocarcinoma.

In this study, transcriptome data and methylation sequencing from The Cancer Genome Atlas (TCGA) were used to examine the immune microenvironment in cholangiocarcinoma. Thirty-six tumor samples were divided into seven subtypes based on cluster analysis. Immune infiltration analysis was performed to screen out “hot” and “cold” tumors from the seven clusters. Finally, ATP2B1 as a prognostic marker for early-warning immune response was determined and verified by immunohistochemistry. The results showed that ATP2B1 is remarkably correlated with patient outcomes in a cohort of 77 patients. Potential targets from the analysis of cold and hot tumors, which can activate immune signals and prompt cold tumor response were explored.

## Materials and Methods

### Clinical Materials

Seventy-seven paraffin-embedded ICC specimens were obtained from 2012 to 2019 at Tianjin Medical University Cancer Institute and Hospital (Tianjin, China). These patients (without any treatment before surgery) underwent curative resection and were pathologically confirmed to have ICC. All specimens were confirmed pathologically by board-certified pathologists (One of our authors, Lisha Qi, is a pathologist who works in the department of pathology in our hospital). Those with adjuvant therapy and lack of follow-up data were excluded. According to the Helsinki declaration, informed consent was obtained for the recruitment of patients. The whole study was approved by the ethics committee.

### RNA-Seq Data Analysis

The gene expression RNA-seq of cholangiocarcinoma was downloaded from TCGA database, including 36 bile duct tumors and 9 adjacent tissues. The RNA-seq expression profiles cholangiocarcinoma (counts and FPKM) were obtained from UCSC Xena, using the gene annotation (gencode.v22.annotation.gtf.gz) for all samples. Transcriptome data of cholangiocarcinoma tumor samples contained 33 cases of ICC, 2 cases of extrahepatic and 1 case of gallbladder cancer.

### Methylation Profiling and Data Analysis

Illumina HumanMethylation_450K array data, including 36 bile duct tumors (33 cases of ICC, 2 cases of extrahepatic and 1 case of gallbladder cancer) and 9 adjacent tissues, were downloaded from UCSC Xena. All raw data were processed using the ChAMP package in R. The probes with more than 10% missing values were not considered in the analysis. The average missing rate of array data was 0.19, and multiple interpolations were performed for the missing values. Furthermore, the probes were filtered if they were not in the CpG context, had known single-nucleotide polymorphisms in the surrounding locus, were aligned to multiple locations in the genome, or were mapped to X and Y chromosomes. Processed methylation data were further normalized using the BETA mixture model BMIQ (10) implemented in the ChAMP package (11), and a BMIQ diagram was drawn. The probe was annotated using the Bioconductor package with hm450.manifest(hg19).

### Correlation Analysis Between DNA Methylation and Genes

Spearson correlation (*r*) was performed to calculate each CpG_gene pair to explore the effect of DNA methylation on the negative regulation of gene expression. r<−0.5 and *P*<0.01 were selected as the cutoff values of significant correlation. The ComplexHeatmap package (12) was used to reveal the patterns and correlations between methylation and gene expression.

### Cluster Analysis

The R software package, consumption cluster plus (13), was used for consistency cluster analysis. The samples were divided into different groups to create a cluster model. An unsupervised consensus clustering of DNA methylation was performed according to the K-means algorithm and Euclidean distance. The PAC method was selected to determine the optimal number of clusters. The Limma package (14) was used to analyze the differences between cluster models. The Gosemsim software package in R was used to identify the key survival-related gene (15).

### Evaluation of TME Score and Immune Infiltration

The Estimate package (16–18) from r-forge was employed to assess the TME, including stromal score, immune score, ESTIMATE score, and tumor purity. Furthermore, according to the median value of the immune scores, cholangiocarcinoma cases were assigned to high and low immune score groups to identify the possible association of immune score with overall survival., Single-sample GSEA (ssGSEA) analysis was carried out to determine the differential changes in 28 immune cell types and further evaluate the changes of immune cell characteristics associated with *ATP2B1* gene.

### Immunohistochemistry (IHC)

Paraffin embedded tissue chips were dewaxed in xylene and alcohol with decreasing concentration gradient. EDTA repair solution (pH = 8) was used for thermal repair to fully expose the antibody, and 3% H_2_O_2_ was used to block endogenous peroxidase. BSA (3%) was blocked at room temperature for 20 min, and antibodies (CD4, 1:400, ab133616; and CD8, 1:200, ab4055) incubated overnight at 4°C after half an hour at room temperature.

The immunohistochemical score classification of ATP2B1 adopts the immune score standard previously studied by our research group (19). The staining intensity of ATP2B1 was divided into 0–3 levels (0 for no staining, 1 for weak immunoreactivity, 2 for medium immunoreactivity, and 3 for strong immunoreactivity). The scoring range of immune response percentage is 0 to 3 (0 for no positive cells, 1 for <30% positive cells, 2 for 30 to 60% positive cells, and 3 for >60% positive cells). The scores of staining intensity and percentage immunoreactivity were multiplied as the final scores of positive staining. We finally classified four expression levels of the staining: negative (score=0); weakly positive (+) (score=1–3); medium positive (++) (score=4–6); and strong positive (+++) (score=7–9). Negative (score = 0) and weakly positive (+) (score = 1 – 3) were defined as low expression; Moderate positivity (+ +) (score = 4 – 6) and strong positivity (+ + +) were defined as high expression.

### Statistical Analysis

Statistical analyses were performed using SPSS 25.0 (IBM Corp., Armonk, NY, USA) and GraphPad Prism (version 8.2.1; San Diego, CA, USA). Wilcoxon test was used for comparison between the two groups. Pearson and Spearman correlation analysis were used to compare the correlation between the two groups of data and calculate the correlation coefficient. Kaplan–Meier curves were calculated using the survival package of R, version 4.1.1. *P* values <0.05 were considered to be significant.

## Results

### Immunological Characterization of Cholangiocarcinoma in Overall Survival

**Figure 1:**
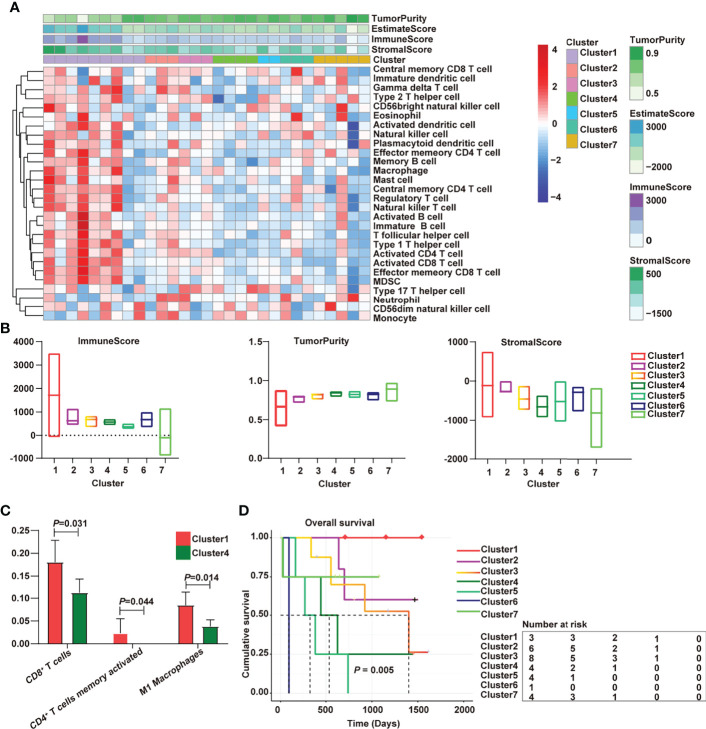
Immunological characterization of cholangiocarcinoma has been implicated in overall survival. **(A)** Heatmap of the immune clusters of cholangiocarcinoma; **(B)** Immune score, stromal score, and tumor purity in the seven clusters; **(C)** Differential immune cells between cold and hot tumors; **(D)** Kaplan–Meier curves of the association of the seven subtypes with overall survival.

The cholangiocarcinoma samples were divided into 7 subtypes by consensus cluster analysis (**Figure S1**). There were significant differences in the distribution of immune cells among 7 subtypes (**Figure 1A**). The “hot” phenotype was enriched in Cluster 1, which includes immune activation cells such as activated dendritic cell, natural killer cell, plasmacytoid dendritic cell, effector memory CD4^+^ T cell, memory B cell, central memory CD4^+^ T cell, natural killer T cell, activated B cell, activated CD4^+^ T cell, activated CD8^+^ T cell, effector memory CD8^+^ T cell, immature B cell, T follicular helper cell, and type 1 T helper cell. T follicular helper cell could activate B cells to promote anti-tumor response (20). Cytotoxic CD8^+^ T cells are the main killers of pathogens and neoplastic cells, and CD4^+^ T cells play remarkable roles in the maintenance of CD8^+^ response and the prevention of exhaustion (21). Cluster 4 was characterized by CD56^dim^ natural killer cells and monocytes and was defined as the “cold” immune phenotype. Additionally, the “hot” phenotype had the highest immune score and the lowest tumor purity (**Figure 1B**). The immune cell components had obvious differences. Compared with cold tumors, the contents of CD8^+^ cells, activated memory CD4 cells, and M1 macrophages were higher in hot tumors (**Figure 1C**). Survival analysis shed light on the relationship between hot immune phenotype and better prognosis (**Figure 1D**). M1 macrophages exhibited pro-inflammatory and antitumor properties (22). The increased number and activation of CD4^+^/CD8^+^ lymphocytes and M1 macrophages are associated with better prognosis (23). These findings are consistent with our results.

### DNA Methylation Regulated Tumor Immunity in Cholangiocarcinoma

**Figure 2:**
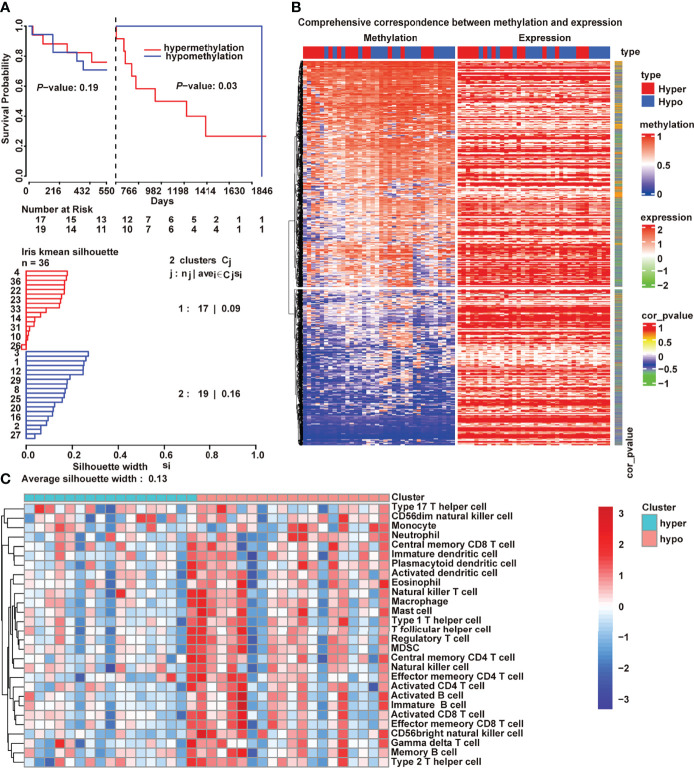
DNA methylation-regulated tumor immunity in cholangiocarcinoma. **(A)** Kaplan–Meier curves of the association of DNA methylation with overall survival; **(B)** Correlation between transcriptome and methylation; **(C)** Differences of 28 immune cells between hypermethylation and hypomethylation.

The regulation of DNA methylation is critical for adaptive immune response, including dendritic cell development andT cell differentiation (24, 25). An unsupervised clustering method was used to assign the samples into DNA hypermethylation and hypomethylation groups to further explore what drives the variations between immunologically hot and cold tumors. In addition, patients which hypermethylation had a worse prognosis than patients with hypomethylation (**Figure 2A**; *P *= 0.03, log-rank test). Methylation deregulation is accompanied by concomitant transcriptome alterations. Thus, we checked the correlation between DNA methylation and mRNA expression (**Figure 2B**) and discovered that 3593 genes (r<−0.5, *P*<0.01) were negatively associated by with DNA methylation in cholangiocarcinoma. Compared with the hypomethylated samples, the hypermethylated cholangiocarcinoma samples had lower tumor immune cell infiltration (**Figure 2C**).

### ATP2B1 Remodels the Tumor Immune Microenvironment

A total of 3313 genes were identified as differential expression genes (DEGs) between hot tumor and cold tumors to explore our immunotyping more deeply (**Supplemental Table 1**). Then, the DEGs and DNA methylation-governed gene expression were intersected, and 1466 genes with high expression and hypomethylation in the hot tumor were obtained.

**Figure 3:**
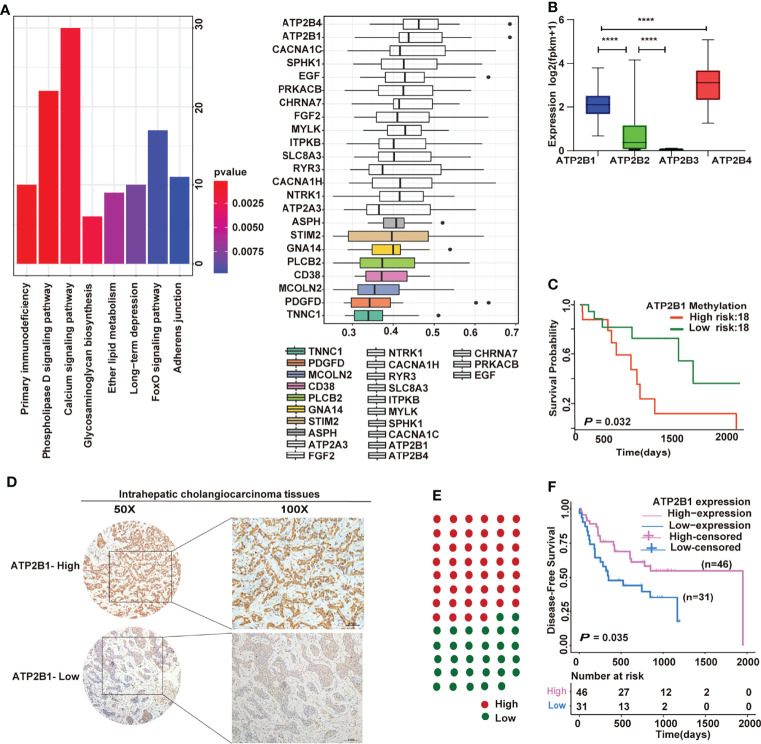
Prognostic significance of ATP2B1 in ICC. **(A)** Pathway enrichment analyses and semantic similarities of gene classes by GOSemSim; **(B)** Expression of four genes in cholangiocarcinoma from TCGA; **(C)** Kaplan–Meier curves of the association of ATP2B1 gene methylation with overall survival; **(D)** Representative IHC staining of ATP2B1 in ICC tissues; **(E)** Proportions of ICC case with different ATP2B1 expression levels; **(F)** Association of ATP2B1 expression with better disease-free survival in ICC.

The results of pathway enrichment analyses are shown in **Figure 3A**. Most of the genes regulated by hypomethylation were enriched in calcium signaling pathways. Next, the Gene Ontology (GO) semantic similarity of these genes were calculated using the GOSemSim package to further mine the key genes. Two genes (*ATP2B4* and *ATP2B1*) that play the most important roles in calcium signaling pathways were obtained. The expression levels of *ATP2B1/4* were compared in cholangiocarcinoma to identify whether the genes were relevant to cholangiocarcinoma progression. Notably, *ATP2B4* expression was dominant over *ATP2B1* (**Figure 3B**), but ATP2B1 hypermethylation predicted a shorter survival time (**Figure 3C**). Importantly, a cohort of 77 cholangiocarcinoma samples was evaluated by IHC staining to further examinate the relationship between ATP2B1 of expression and patient prognosis. The representative micrographs of different ATP2B1 expression levels are shown in **Figure 3D**. Among the patients, about 60% had positive ATP2B1 staining (**Figure 3E**). A correlation between ATP2B1 expression and the ICC prognosis was found, and further validated by the IHC staining of clinical samples (**Figure 3F**).

**Figure 4:**
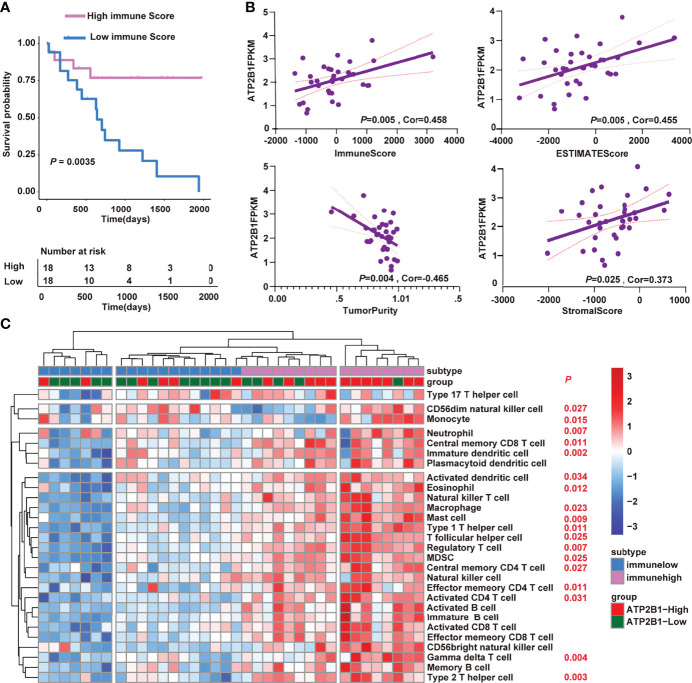
ATP2B1 remodeling the tumor immune microenvironment. **(A)** Kaplan–Meier curves of the correlation of immune score with the prognosis of overall survival; **(B)** Correlation between ATP2B1 expression and the tumor immune microenvironment; **(C)** Differences of 28 immune cells in the high and low ATP2B1 expression groups.

Next, we tested whether ATP2B1 expression is associated to changes in TME and is a prognostic factor for better survival. First, we discovered the effect of immune score on the prognosis of cholangiocarcinoma. A higher immune score corresponds to a longer overall survival time (*P*=0.0035, **Figure 4A**). Furthermore, we found a strong association between ATP2B1 expression and TME composition (**Figure 4B**). The tumor tissues with a relatively high ATP2B1 expression contained more immune stromal components and had a higher ESTIMATE score. The correlations between TME components and ATP2B1 expression was by Pearson’s correlation analysis. ATP2B1 expression was significantly positively correlated with immune scores (*P*=0.005, r=0.458), stromal scores (*P*=0.025, r=0.373) and ESTIMATE scores (*P*=0.005, r=0.455). ATP2B1 was negatively correlated with tumor purity (*P*=0.004, r=-0.465). In addition, the profiles of 28 infiltrating immune cells based on *ssGSEA* score were revealed in different groups (**Figure 4C**). The majority of the ATP2B1 high-expression-group assembled in high immunity, indicating a significant increase in immune infiltration, and 18 out of 28 immune cells showed remarkable differences compared with low-expression group (*P*<0.05). In summary, the *ATP2B1* gene enabled the accurate identification of immune cells that changed in the remodeling of the immune microenvironment.

### Relationship Between ATP2B1 and Immune Cells

**Figure 5:**
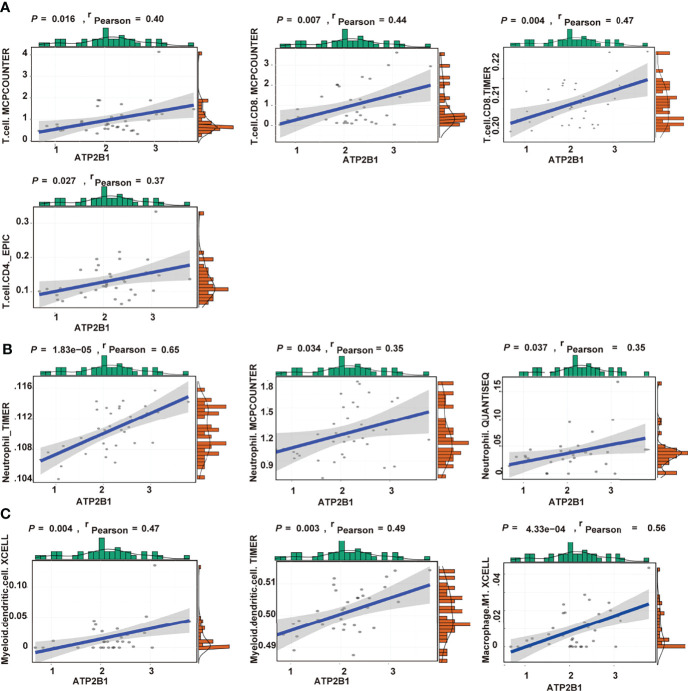
Association of CD8+T cells, CD4+T cells **(A)**, neutrophil **(B)**, macrophage and myeloid dendritic cells **(C)** with ATP2B1 expression.

From the above results, we concluded that ATP2B1 expression can be accompanied by changes in immune cells. The correlation between gene expression and immune cells was further analyzed. Finally, the levels of immune cells, including CD8^+^ T cells, CD4^+^ T cells, macrophage, neutrophils and myeloid dendritic cells that were closely associated with ATP2B1 expression were identified. Positive correlations were found between ATP2B1 expression and the levels of infiltrating T cells (*P*=0.016, r=0.40; **Figure 5A**). The level of CD8^+^ T cell infiltration had the highest correlation with ATP2B1 expression (*P*=0.004, r=0.47). The correlations between ATP2B1 expression and the levels of CD8^+^ and CD4^+^ T cells were diverse but positive. Moreover, data from TIMER, MCPCOUNTER, and QUANTISEQ confirmed the positive correlation between ATP2B1 expression and neutrophil infiltration (**Figure 5B**). Myeloid dendritic cell and M1- macrophages were also positively correlated with ATP2B1 expression (**Figures 5C, D**).

**Figure 6:**
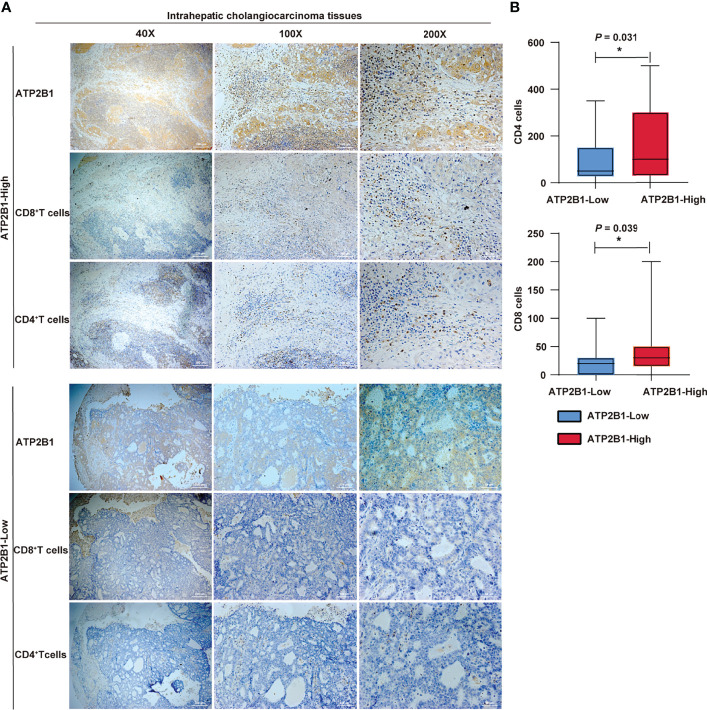
*ATP2B1* expression in CD4^+^ and CD8^+^ immune cells. **(A)** IHC staining analysis of CD4^+^ and CD8^+^ immune cells; **(B)** Numbers of CD4^+^ and CD8^+^cells detected by IHC. *P < 0.05; **P < 0.01; ***P < 0.001.

The results between ATP2B1 expression and T cell infiltration was contrasted to assess whether ATP2B1 expression at the protein level can recruit immune cells. The results showed that the levels of CD4^+^ and CD8^+^ T cells in cholangiocarcinoma with ATP2B1 protein overexpression were higher under different magnifications (**Figure 6A**). Next, the expression difference between the numbers of infiltrating CD8^+^ and CD4^+^ T cells were evaluated according to ATP2B1 expression and found that the upregulation ATP2B1 was correlated with higher levels of CD4^+^ T cells (*P*=0.031, **Figure 6B**) and CD8^+^ T cells (*P*=0.039, **Figure 6B**).

## Discussion

ICC is one of the most invasive tumors in which the immunotherapies or standard therapies alone are mostly ineffective owing to its complex immune microenvironment. Intensive studies of the TME in recent years has confirmed the different immune subtypes related to distinct patient survival (26, 27). A multi- omic analysis of ICC revealed that immune classification is a remarkable independent factor of relapse-free survival, and the high-immune group has better prognosis than the low-immune group (28). Moreover, the high-immune subtype with abundant adaptive immune cell (T and B cells) infiltrates achieved better immunotherapy response when treated with anti-PD-1 immunotherapy. In addition, upregulated immune pathways and a higher expression of the IFN signature were observed in the high-immune subtype (24, 28). Accordingly, these tumors were characterized by adaptive immune-resistance mechanisms, whereas, low-immune subtype was immunological ignorant.

The expression fold change of markers between the “hot” and “cold” immune types were compared o explore the treatment strategy of transforming immune cold tumor into immune hot tumor. The results showed that ATPase plasma membrane Ca^2+^ transporting-1 (ATP2B1 or PMCA1) plays an important role in the prognosis of cholangiocarcinoma. ATP2B1 expression has been reported to be correlated with the progression of various types. The ATP2B1 contents of extracellular vesicles are increased in prostate cancer treated with enzalutamide and are negatively regulated by androgen receptor (29). In comparison with the downregulation of ATP2B1 expression in oral cancer (30), ATP2B1 expression levels are upregulated in breast cancer. Interestingly, the absence of ATP2B1 considerably increases the efficacy of ionomycin in breast cancer (31). Furthermore, ATP2B1 overexpression is associated with cisplatin resistance in human ovarian adenocarcinoma (32). Jiang et al. also identified an increased expression of the *ATP2B1* gene, which encodes a calcium pump, in the monocytes of patients with acute respiratory distress syndrome (33).

Although it is limited to understanding intracellular Ca^2+^ homeostasis in cancer. Accumulating evidence suggests that the proteins involved in Ca^2+^ transport may be potential therapeutic targets. In prostate cancer, TRPM8 antagonists, which perturb the androgen-elicited rapid responses, lower intracellular calcium levels and inhibit cell proliferation (34). TRPV1 overexpression increases Ca^2+^ and causes CaMKKβ activation and AMPK phosphorylation, which leads to the suppression of GC cell proliferation, migration, and invasion (35). TRPV6 knockdown or TRPV6 inhibitor can inhibit the proliferation, migration and invasion of cancer cells by regulating calcium signal activation, which has proved to be effective in the treatment of breast cancer, ovarian cancer, prostate cancer and pancreatic cancer (36, 37). In addition, the study on purinergic P2 receptor (induces intracellular Ca^2+^ increases) in pancreatic ductal adenocarcinoma confirmed that the combination of AR-C118925XX, a selective antagonist of P2RY2 receptor, and gemcitabine had a synergistic effect, prolonging the survival time of xenotransplantation PDAC mice (38). Previous studies have found that SERCA (sarcoplasmic/endoplasmic reticulum Ca2+-ATPase) inhibitors (curcumin and mipsagargin) promoted the apoptosis of tumor cells. To sum up, abnormal calcium balance is closely related to the malignant progression of tumors. Although the housekeeping form of ATP2B1 (PMCA1) is stable during development, its expression was changed in oral cancer and breast cancer (30, 31). As a calcium pump protein, ATP2B1 dysfunction may lead to the progression of CCA. In our study, ATP2B1 overexpression was strongly correlated with better ICC prognosis. ATP2B1 may be used as a marker to predict the prognosis of ICC. Moreover, targeting ATP2B1 to regulate calcium homeostasis may be a new therapeutic strategy for the prognosis of ICC.

Our study identified ATP2B1-mediated changes in the TME. Immune cells are key players in the liver cancer microenvironment. The effector function of immune cells as well as the proliferation and apoptosis of cancer cells, depends on Ca^2+^ signaling (39). When lymphocytes are activated, plasma membrane calcium release-activated channels (CRACs) are triggered to open (40). Cytoplasmic Ca^2+^ concentration remarkably increases with the influx of extracellular Ca^2+^. If the intracellular Ca^2+^ concentration is high to a certain extent, it will inhibit the opening of the CRAC and restrict further calcium influx. Thus, the activation of lymphocytes and other immune cells requires calcium efflux channels. ATP2B1 is localized into membrane compartments and exports Ca^2+^. ATP2B1 upregulation enhances Ca^2+^ clearance from the stimulated cells. TRPM4 couldn’t be activated; thus, Ca2+ influx occurs *via* CRAC. The influx of extracellular Ca^2+^ is essential for activating NF-kB signaling, which promotes the M1 polarization of tumor-associated macrophages (41). ATP2B1 is expressed in T cells and increases following T cell activation (42, 43). ATP2B1 overexpression, which increased considerably in NFAT activity, was observed in activated Jurkat T cells (44). Furthermore, ATP2B1 knockout mice have greatly reduced B cell counts (45). These studies proved that ATP2B1 is closely related to lymphocyte and macrophage activation. In addition, M1-like macrophages displayed tumoricidal effects by producing ROS, activating effector T cells, and promoting cell apoptosis.

We performed correlation analysis and found a positive correlation between ATP2B1 and immune cells, especially CD4^+^ and CD8^+^ T cells, in pathological tissues. The results suggest that the modulation of Ca^2+^ signaling by enhanced ATP2B1 upregulation may transform intrahepatic cholangiocarcinoma from cold tumor to hot tumor and increase the efficacy of immunotherapy. It is expected to become a new drug target and an attractive anti-cancer clinical treatment tool. However, the molecular mechanism of ATP2B1 in regulating tumor immune microenvironment needs to be further studied both *in vivo* and *in vitro*.

## Conclusions

In conclusion, we revealed ATP2B1 can be a prognostic factor for cholangiocarcinoma. Upregulation of ATP2B1 in immune cold tumors increases the level of immune cell infiltration, further activates immune signals and induces immune response.

## Data Availability Statement

The original contributions presented in the study are included in the article/**Supplementary Material**. Further inquiries can be directed to the corresponding authors.

## Ethics Statement

The studies involving human participants were reviewed and approved by Ethics Committees of Tianjin Medical University Cancer Institute and Hospital. The patients/participants provided their written informed consent to participate in this study.

## Author Contributions

HG and WL designed the research studies; XZ, YH, LC, ZH, LQ, LC, and YL collected data and analyzed; XZ and YH analyzed the clinical data; YH, LC, and YL collected clinical samples; LQ assessed Immunohistochemistry staining score; HG helped with the retouching of the article language; XZ wrote the manuscript; HG and WL revised the manuscript. All authors read and approved the final manuscript.

## Funding

I am very grateful to the financial support from Natural Science Foundation of China (82173208, 82103672).

## Conflict of Interest

The authors declare that the research was conducted in the absence of any commercial or financial relationships that could be construed as a potential conflict of interest.

## Publisher’s Note

All claims expressed in this article are solely those of the authors and do not necessarily represent those of their affiliated organizations, or those of the publisher, the editors and the reviewers. Any product that may be evaluated in this article, or claim that may be made by its manufacturer, is not guaranteed or endorsed by the publisher.

## Supplementary Material

Supplementary Figure 1 Sample clustering heatmap.
