# Supplementary figures and images for: Low expression and Hypermethylation of ATP2B1 in Intrahepatic Cholangiocarcinoma Correlated With Cold Tumor Microenvironment
Source: Front Oncol. 2022 Jul 7;12:927298. doi: 10.3389/fonc.2022.927298 (PMC9302110; doi:10.3389/fonc.2022.927298)

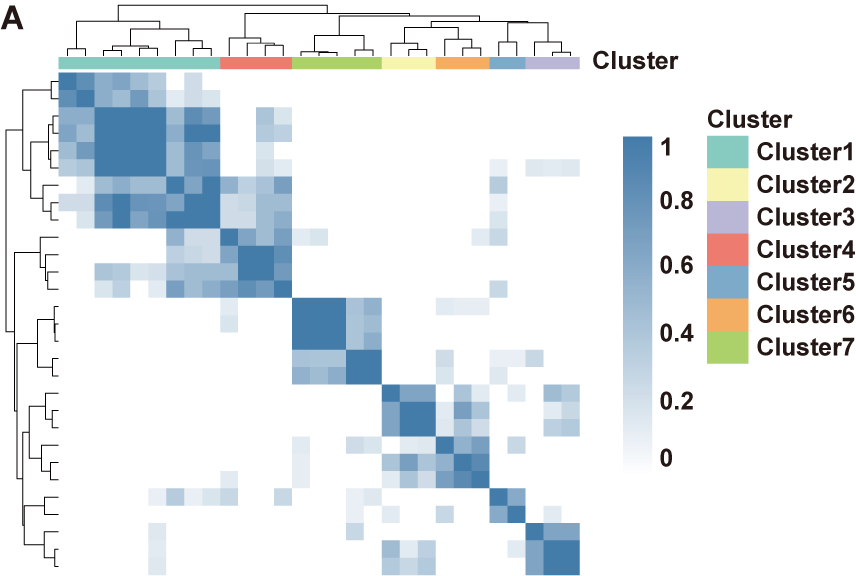

Supplement: Supplementary Figure 1 — Sample clustering heatmap. [file Image_1.tif]
